# Using PacBio SMRT Sequencing Technology and Metabolomics to Explore the Microbiota-Metabolome Interaction Related to Silage Fermentation of Woody Plant

**DOI:** 10.3389/fmicb.2022.857431

**Published:** 2022-06-20

**Authors:** Zhumei Du, Lin Sun, Yanli Lin, Fuyu Yang, Yimin Cai

**Affiliations:** ^1^College of Grassland Science and Technology, China Agricultural University, Beijing, China; ^2^Japan International Research Center for Agricultural Sciences (JIRCAS), Tsukuba, Japan; ^3^Inner Mongolia Academy of Agricultural Sciences and Animal Husbandry, Hohhot, China; ^4^Beijing Sure Academy of Biosciences, Beijing, China

**Keywords:** exogenous additive, metabolites, microbiota, silage fermentation, woody plant

## Abstract

Silage fermentation is a dynamic process involving the succession of microbial communities and changes in metabolites. Fresh branched and leaves of paper mulberry were used to prepared silage. Crop by-products including corn bran, rice bran, and wheat bran were used as exogenous additives. Pacific Biosciences single-molecule real-time (SMRT) sequencing technology and metabolomics are used to explore the interaction mechanism of microbial structure and metabolites during woody silage fermentation and to verify the principle that exogenous additives can modulate silage fermentation. Under the dual stress of anaerobic and acidic environment of silage fermentation, the microbial community changed from Gram-negative to Gram-positive bacteria, and a large amount of lactic acid and volatile fatty acid were produced, which lowered the pH value and caused the rapid death of aerobic bacteria with thin cell walls. The exogenous additives of corn bran and wheat bran accelerated the dynamic succession of lactic acid bacteria as the dominant microbial community in silage fermentation, increased the metabolic pathways of lactic acid, unsaturated fatty acids, citric acid, L-malic acid and other flavoring agents, and inhibited the growth of *Clostridium* and *Enterobacter*, thereby improving the flavor and quality of the silage. However, because rice bran contained butyric acid spore bacteria, it can multiply in an anaerobic environment, led to butyric acid fermentation, and promoted protein degradation and ammonia nitrogen production, thereby reduced the fermentation quality of woody silage. The results showed that during the silage fermentation process, the microbial community and the metabolome can interact, and exogenous additives can affect the fermentation quality of silage. SMRT sequencing technology and untargeted metabolomics revealed the microbiota-metabolome interaction during silage fermentation. Changes in the structure of the microbial community can affect the metabolic pathways, and the final metabolites can inhibit the growth of microorganisms that are not conducive to silage fermentation. Exogenous carbohydrate additives can change the fermentation substrate and affect microbial community structure, thus modulate the silage fermentation.

## Highlights

- Microbiota influence the metabolite profile and fermentation quality of silage.
- Metabolites affect microbial community structure and silage flavor.
- Exogenous additives affect the microbiota and metabolome of silage.
- Brans modulate the production of amino acids and unsaturated fatty acids in silage.
- SMRT and metabolomics accurately reveal the microbiota–metabolome interaction.

## Introduction

In tropical developing countries, the main sources of roughage for ruminants are native grasses and crop by-products (Cai et al., 2020). With the growing population and decreasing area of arable land, conventional feeds, such as forage crops, grasses, and grains, cannot meet the demands of ruminants. The development of new feed resources is important for promoting animal production, improving people’s livelihood, and achieving Sustainable Development Goals (SDGs; Keeling et al., 2019). Paper mulberry [PM, *Broussonetia papyrifera* (L.) Vent] is a deciduous tree or shrub in the family *Moraceae*. Due to its high nutritional value and antioxidant and antibacterial bioactive ingredients, the fresh branches and leaves of PM are a potential feed resource in the tropics. PM can adapt to arid climates and various soil conditions; its high biomass yield and low feed cost can increase farmers’ profits. Woody plant (WP) can be used as a feed source for ruminants, which is of great significance for alleviating feed shortages and promoting animal production.

PM is generally harvested in the rainy season. If prepared and preserved like hay, large amounts of nutrients will be lost, therefore, silage is considered as an ideal preparation method (Du et al., 2021a). In the previous study (Du et al., 2021a), it has been reported that microbial additives such as lactic acid bacteria (LAB) inoculant and cellulolytic enzyme have a good improvement effect on WP silage fermentation. In addition, in order to improve the fermentation quality, silage is often prepared with agricultural by-products including brans, but information of WP silage prepared with bran has not been reported. Silage fermentation is a very important feed storage method. It usually involves the microbial fermentation of fresh grasses in an anaerobic environment in a silo. Silage fermentation is a dynamic process involving microbial community succession and metabolite changes (Ding et al., 2013). The forage materials provide microorganisms with fermentation substrates, such as water-soluble carbohydrate (WSC), which the microorganisms used to produce organic acids, lowering the pH, and enabling the long-term preservation of silage. During silage fermentation, the microbial community interacts with metabolites, affecting the fermentation quality of silage. More than 20% of the total silage produced annually is lost *via* preparation failure (Borreani et al., 2018). Therefore, it is important to explore the mechanisms modulating silage fermentation to produce high-quality feed.

Forage moisture, WSC, and epiphytic LAB are important factors affecting silage fermentation. Corn bran (CB), rice bran (RB), and wheat bran (WB) are common agricultural by-products in livestock production, which are characterized by low moisture, high WSC and rich nutrient. To overcome the influences of the unfavorable factors on silage fermentation, bran addition is also one of the widely used silage preparation techniques. This study used three crop by-products (CB, RB, and WB) as exogenous additives to optimize silage fermentation by adjusting the fermentation substrate. Pacific Biosciences (PacBio) single-molecule real-time (SMRT) sequencing technology combined with metabolomics was used to explore the succession and diversity of microbial communities at the species level, and interactions between the microbiota and metabolome, to establish a theoretical basis for controlling the fermentation process and preparing high-quality silage.

## Materials and Methods

### Silage Preparation of PM

The PM hybrid (*Broussonetia kazinoki* × *B. papyrifera*) used in this study was cultivated at the mountain experimental field of Guizhou Qianchang Shenghe modern agriculture Co., Ltd. (106^o^37′E longitude and 25^o^73′N latitude, Daihua, Guizhou, China). The station had a mid-subtropical monsoon humid climate with a mean annual temperature of 15.5°C (extreme maximum temperature is 34°C–36°C, minimum temperature is −6°C to −9°C), annual relative humidity of 81%, and annual rainfall of 1,250–1,400 mm. The growth height of PM is about 1.8 m, and it is harvested 4–5 times a year, leaving 20 cm of stubble each time. Fresh young shoots and leaves were collected from the second cutting, and three field replicates were performed on 12 June 2020.

The CB, RB, and WB materials were purchased from Guilian Animal Feed Co., Ltd. (Guiyang, China). First use a chopper (92-2S, Sida Agri-Machine Co., Ltd., Luoyang, China) to cut PM into a length of 1–2 cm, and then silages were prepared from PM unmixed. The mixture silages were also prepared from PM and brans (CB, RB, or WB) at a percentage of 70 + 30, based on the fresh matter (FM). Approximately 20 kg of PM and mixture materials for each treatment were packed into the polyethylene drum silos (30 L, Huafang Plastic Co., Ltd., Jiangsu, China) with three replicates, air in the material was tightly compressed and discharged, and then sealed with a cover to maintain anaerobic conditions. The silos were stored in a ventilated warehouse at ambient temperature (21°C–35°C). After 128 days of fermentation, the silages were opened, and the samples of each treatment were divided into three parts for microbial and chemical analysis. The fresh parts were immediately collected in a sterilization bag and kept in an icebox, and then immediately transported to the laboratory for analysis of microbial population and chemical composition. At the same time, samples for analyzing microbial community and metabolites are quickly stored in a freezer at −80°C for future extraction of DNA and liquid.

### Analysis of Chemical Composition, Silage Fermentation, and Microbial Population

The dry matter (DM), organic matter (OM), crude protein (CP), ether extract (EE), neutral detergent fiber (NDF), acid detergent fiber (ADF), and acid detergent lignin (ADL) were analyzed in accordance with the methods of the Association of Official Analytical Chemists (AOAC International, 2000). WSC, pH, ammonia nitrogen (NH_3_-N) and organic acids were determined in accordance with methods as described by Cai (2004). The pH was determined by a pH meter (D-71, Horiba, Kyoto, Japan). The NH_3_-N content was measured using the Kjeltech auto distillation system (2200, Foss Tecator, Hoganas, Sweden). The organic acid was analyzed by a high-performance liquid chromatography system (HPLC, LC-2000, Jasco, Tokyo, Japan) by following conditions: detector, UV-2070 (JASCO Corporation, Tokyo, Japan), 450 nm; column, Shodex RS pak KC-811 (8.0 mm 30 cm; Shoko, Tokyo, Japan); oven temperature, 60°C; eluent, 3 mmol/L HClO_4_; flow rate, 1.0 ml/min. Lactic acid buffer capacity (LBC) was analyzed by a titration method (Muck et al., 1991).

The microbial population was determined by a plate count method as described by Cai (2004). The LAB, aerobic bacteria, coliform bacteria and yeasts (including molds) were analyzed by the mediums of de Man, Rogosa, and Sharpe agar (Difco Laboratories, Detroit, MI, United States), nutrient agar (Nissui-Seiyaku Co., Ltd., Tokyo, Japan), blue light broth agar (Nissui-Seiyaku Co., Ltd.) and potato dextrose agar (Nissui-Seiyaku Co., Ltd.), respectively. LAB were incubated in an anaerobic box (ANX-1; Hirosawa Ltd., Tokyo, Japan) at 30°C for 2–3 days, and other microorganisms were in an anaerobic incubator at 30°C for 2–5 days. The growth status of microbial colonies were observed every day during the incubation period, and the colonies were counted as viable numbers in colony-forming unit per gram (cfu/g) of FM.

### Microbial Community Analyses

For SMRT sequencing analysis, the triplicate samples (10 g) were mixed with 90 ml of sterilized 0.85% NaCl solution and shaken at the speed of 250 rpm in a 4°C refrigerator for 45 min. The liquid mixture was filtered through a four-layer cheesecloth pre-autoclaved, and then the filtrate was centrifuged at 10,000 rpm for 10 min at 4°C to obtain microbial precipitate (Du et al., 2021b). Total genomic DNA from each sample was extracted using the TIANamp Bacteria DNA isolation kit (DP302-02, Tiangen, Beijing, China). The quality of the extracted DNA was assessed in 1% agarose gels using electrophoresis and spectrophotometry (optical density at 260/280 nm ratio). All DNA samples were purified through a DNA kit column and stored at −20°C until further analyses (Hou et al., 2015; Du et al., 2021b).

The PCR product of the full length of 16S rRNA regions were amplificated as described by Du et al. (2021b). The 16S rRNA gene was amplified by PCR for SMRT sequencing using the 27F (AGRGTTTGATYNTGGCTCAG) and 1492R (TASGGHTACCTTGTTASGACTT) primers. The PCR program was: 95°C for 5 min; 30 cycles of 95°C for 30 s, 50°C for 30 s, and 72°C for 60 s, with a final extension of 72°C for 7 min. The Agencourt AMPure XP Beads (Beckman Coulter, Indianapolis, IN) were used for purification of the total of PCR amplicons. The Qubit dsDNA HS Assay Kit and Qubit 3.0 Fluorometer (Invitrogen, Thermo Fisher Scientific, Oregon, United States) was used for quantification analysis. Library construction used PacBio SMRTbell™ Template Prep Kit 2.0 (Menlo Park, CA, United States) on normalized pooled PCR products (Earl et al., 2018). The amplicons were sequenced on a PacBio RS II platform (Pacific Biosciences) using P6-C4 chemistry (Hou et al., 2015). The quality control of PCR amplifications and sequence pre-processing were performed according to the method described by Mosher et al. (2013).

Raw data were processed using the protocol RS_Readsofinsert.1 available in SMRT Portal version 2.7 software (PacBio). Firstly, the high-quality sequences were performed using the Quantitative Insights In to Microbial Ecology (QIIME) package (version 1.7.0; Caporaso et al., 2010). Under 100% clustering of sequence identity, using the analyses of Python nearest alignment space termination (PyNAST) and clustering and classification inference with U-statistics (UCLUST) to align the extracted high-quality sequences to obtain representative sequences (Edgar, 2010). After the selection of the representative sequences, the unique sequences were classified into operational taxonomic unit (OTU) based on 99% threshold identity using the UCLUST algorithm (Lozupone and Knight, 2005). Potential chimeric sequences in the representative set of OTU were removed using the Chimera Slayer tool (Haas et al., 2011). According to the analysis of the OTU clustering, and the research requirements to analyze both the core and unique information for material and silage samples. The SILVA database version 132 was implemented to classify different OTU and annotate the taxonomic information for each OTU representative sequence based on Bergey’s taxonomy at the genus, family, order, class, and phylum levels, according to classification at an 80% minimum bootstrap threshold (Quast et al., 2013). OTU that occurred only once or twice were discarded. Unweighted Pair-group Method with Arithmetic Means (UPGMA) clustering was performed to use average linkage to explain the distance matrix in QIIME software (version 1.7.0; Chen et al., 2020).

### Untargeted Metabolomics Profiling Analysis

The silage sample (5 g) was placed in 20 ml of cold extraction liquid (methanol:water = 4:1) for extraction in a microcentrifuge tube. Each treatment performs six biological replicates for metabolomic profiling detection analysis. The untargeted metabolomics analyses were carried out by ultra HPLC tandem time-of-flight mass spectrometry (UHPLC/TOF-MS). The liquid chromatography with mass spectrometry (LC–MS) untargeted metabolomics analyses were performed on an Agilent 1100 series HPLC system (Agilent Technologies, Palo Alto, CA, United States) with a UPLC BEH Amide column (1.7 μm, 2.1 mm × 100 mm, Waters, Milford, United States) coupled to Triple TOF 5600 (Q-TOF, AB Sciex, Framingham, Massachusetts, United States). The detailed information of the UHPLC/TOF-MS program was following the previous study by Wang et al. (2019). In order to evaluate the stability of the analytical system during the determination process, every five samples were designed as a quality control (QC) sample.

### Metabolomic Data Pre-processing

Before statistical analysis, the files of LC–MS raw data were converted for pre-processing by the software of Progenesis QI (version 2.0, Waters Corporation, Milford, MA, United States). The result of pre-processing generates a data matrix with the following contents: baseline filtration, peak identification, integration, retention time correction, peak alignment, and finally a data matrix of retention time, mass-to-charge ratio, and peak intensity. In all the samples, variables with non-zero values more than 50% were retained; the missing values with one-half of the minimum value in the original matrix were filled; the total peaks were normalized, and the QC samples with relative standard deviation (RSD) ≥ 30% were deleted. The LC-MS analytical system had appropriate stability and repeatability and that the acquired data were sufficient quality for subsequent determination.

### Identification of Metabolites

The metabolite annotation was performed by software Progenesis QI (version 2.0, Waters Corporation, Milford, MA, United States), integrated with commercial databases of human metabolome database (HMDB). Based on the detected peaks, the metabolites were identified by the interquartile range denoising method. Missing raw data values were set to half of the minimum value of the detection limit. In addition, an internal standard normalization method was used in these data analyses.

According to metabolite clustering, the Venn diagram package (version 1.2) of R packages statistical tools was draw (Shade and Handselman, 2012). The value of variable importance in projection (VIP) score was analyzed by ropls (R packages, version 1.6.2) and scipy (Python, version 1.0.0; Chong et al., 2018). The principal component analysis (PCA), partial least-square discriminant analysis (PLS-DA), and orthogonal partial least squares discriminant analysis (OPLS-DA) were analyzed by the ropls (R packages, version 1.6.2) software package (Wehrens and Mevik, 2007). Input data were the total mass of the signal integration area of each sample, and the signal integration area was normalized with method of internal standard normalization for each sample. Correlation analyses was also performed using a Python-based tool (Langfelder and Horvath, 2008).

### Statistical Analyses

The ANOVA was performed using the general linear model (GLM) procedure of Statistical Package for the Social Sciences (SPSS version 19.0, SPSS Inc., Chicago, IL, United States) to examine the differences between samples, and significance was declared at *p* < 0.05. The LBC, chemical composition and fermentation quality data for the materials and silages were subjected to one-way ANOVA. Tukey’s honest significant difference test (HSD) was employed for different sample means (Steel and Torrie, 1980).

Welch’s two-sample T-test was used to identify biochemicals that differed significantly between experimental groups. We defined significantly different compounds between treatment groups by the criteria of VIP > 1 and *p* < 0.05. The correlation analyses of the bacterial community with metabolites with biofunctional activity at species level, respectively. The metabolites are displayed horizontally, respectively, and the bacterial community information is displayed vertically. The corresponding value of the middle heat map is the Spearman correlation coefficient r, which ranges between −1 and 1, *r* < 0 indicates a negative correlation (blue), *r* > 0 indicates a positive correlation (red), and “*,” “**,” and “***” represent *p* < 0.05, *p* < 0.01, and *p* < 0.001, respectively.

## Results

The LBC, microbial population, and chemical composition data of all assessed materials are shown in Table 1. The LBC content was higher (all *p* < 0.001) in PM than in the CB, RB, and WB materials. The LAB, coliform bacteria, and yeast counts in PM were 10^3^ to 10^4^ cfu/g of FM, but they were below the detectable level (10^2^) in CB, RB, and WB. The aerobic bacteria counts were 10^3^–10^5^, and the mold counts were below the detectable level (10^2^) in all materials. The moisture content in PM was >80%, but it was <6% in CB, RB, and WB. The CP and ADF contents were higher (all *p* < 0.001) in PM than in CB, RB, and WB, whereas the OM and WSC contents showed the opposite pattern. The EE content in RB and the NDF content in WB were higher (all *p* < 0.01) than in the other materials.

**Table 1:** LBC, microbial population, and chemical composition data of all assessed material.

Item	Material^1^				SEM	*p*
	PM	CB	RB	WB		
LBC (mEq/kg of DM)^2^	892.27a	333.93b	362.02b	347.15b	20.46	<0.001
Microbial population (cfu/g of FM)^3^						
Lactic acid bacteria	4.6 × 10^4^	ND	ND	ND	–	–
Aerobic bacteria	2.7 × 10^5^	2.4 × 10^3^	3.1 × 10^3^	4.1 × 10^3^	–	–
Coliform bacteria	3.1 × 10^3^	ND	ND	ND	–	–
Yeast	2.5 × 10^4^	ND	ND	ND	–	–
Mold	ND	ND	ND	ND	–	–
Chemical composition^4^						
DM (%)	18.75c	94.77b	97.90a	96.00a	0.83	<0.001
OM (% of DM)	91.80d	98.60a	96.40b	95.93c	0.25	<0.001
CP (% of DM)	24.65a	20.28b	14.00d	17.93c	0.35	<0.001
EE (% of DM)	4.55b	3.76c	11.11a	4.55b	0.12	<0.001
NDF (% of DM)	37.57b	40.84b	22.73c	51.07a	1.84	<0.001
ADF (% of DM)	18.52a	15.71c	17.40b	15.31c	0.40	0.0015
WSC (% of DM)	4.14b	8.04a	8.50a	8.94a	0.05	<0.001

^a–d^Data are means of three samples, means in the same row followed by different letters differ (*p* < 0.05).

1 PM, paper mulberry; CB, corn bran; RB, rice bran; and WB, wheat bran.

2 LBC, lactic acid buffer capacity and DM, dry matter.

3 FM, fresh matter and ND, not detected.

4 OM, organic matter; CP, crude protein; EE, ether extract; NDF, neutral detergent fiber; ADF, acid detergent fiber; and WSC, water-soluble carbohydrate.

The microbial population, fermentation quality, and chemical composition of silages are shown in Table 2. The LAB counts were 10^7^ cfu/g of FM in the PM + CB (PC) and PM + WB (PW) silages; these values were higher (all *p* < 0.001) than in the PM and PM + RB (PR) silages. The aerobic bacteria counts were 10^4^–10^5^ in all the silages. The counts of coliform bacteria and mold in the PR silages were 10^3^ and 10^2^, respectively; they were below the detectable level (10^2^) in other silages. The yeast counts were 10^2^–10^3^ in PC, PR, and PW, but this value was below the detectable level in PM silage. The pH values were > 5.07 in the PM and PR silages, whereas they were < 4.14 in the PC and PW silages. Compared with the PM silage, the PC and PW silages had higher (both *p* < 0.001) lactic acid content and lower (all *p* < 0.001) acetic acid and NH_3_-N contents. The propionic acid and butyric acid contents were higher (both *p* < 0.001) in the PR silage than in the PM silage, whereas these values in other silages were below the detectable level (0.001%). The DM contents were below 36% in the PC, PR, and PW silages; these values were higher (all *p* < 0.001) than the value in PM silage. The OM content showed the opposite pattern. The EE content was higher (all *p* < 0.001) in PR silage than in PM, PC, and PW silages.

**Table 2:** Microbial population, fermentation quality, and chemical composition of silage.

Item	Silage^1^				SEM	*p*
	PM	PC	PR	PW		
Microbial population (cfu/g of FM)^2^						
Lactic acid bacteria	3.1 × 10^6^	4.5 × 10^7^	3.4 × 10^6^	4.2 × 10^7^	–	–
Aerobic bacteria	5.4 × 10^4^	4.9 × 10^4^	7.0 × 10^5^	3.0 × 10^4^	–	–
Coliform bacteria	ND	ND	4.2 × 10^3^	ND	–	–
Yeast	ND	2.4 × 10^2^	3.5 × 10^3^	5.9 × 10^2^	–	–
Mold	ND	ND	2.8 × 10^2^	ND	–	–
Fermentation quality^3^						
pH	5.11a	4.08c	5.07b	4.14c	0.08	<0.001
Lactic acid (% of FM)	0.56b	1.44a	0.49c	1.46a	0.04	<0.001
Acetic acid (% of FM)	0.38b	0.14c	0.46a	0.13c	0.01	<0.001
Propionic acid (% of FM)	0.12b	ND	0.21a	ND	0.01	<0.001
Butyric acid (% of FM)	0.33b	ND	0.38a	ND	0.03	<0.001
NH_3_-N (g/kg of FM)	0.23b	0.15d	0.31a	0.18c	0.01	<0.001
Chemical composition^4^						
DM (%)	17.91c	34.82b	35.97a	34.75b	0.30	<0.001
OM (% of DM)	91.81b	93.49a	93.50a	93.72a	0.13	<0.001
CP (% of DM)	24.39b	25.49ab	24.41ab	25.62a	0.33	0.10
EE (% of DM)	4.76b	4.42b	5.35a	4.69b	0.09	0.0014
NDF (% of DM)	36.29b	37.81a	36.26b	38.50a	0.36	0.0048
ADF (% of DM)	18.37	8.30	18.39	18.24	0.15	0.88

^a–d^Data are means of three samples, means in the same row followed by different letters differ (*p* < 0.05).

1 PM, paper mulberry; CB, corn bran; RB, rice bran; WB, wheat bran; PC, PM + CB; PR, PM + RB; PW, PM + WB.

2 FM, fresh matter and ND, not detected.

3 NH_3_-N, ammonia nitrogen.

4 DM, dry matter; OM, organic matter; CP, crude protein; EE, ether extract; NDF, neutral detergent fiber; and ADF, acid detergent fiber.

**Figure 1:**
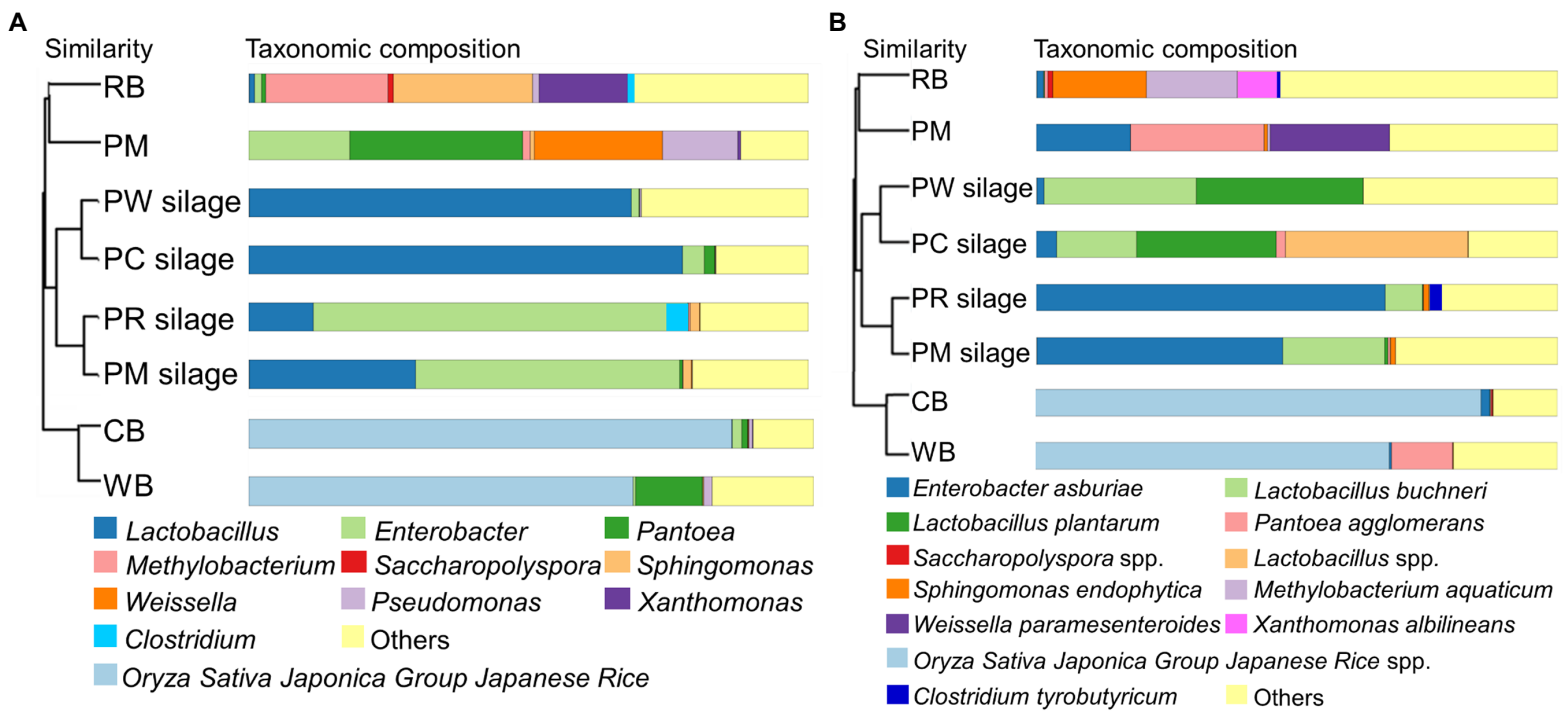
UPGMA hierarchical cluster tree based on the weighted uniFrac distance at the genus **(A)** and species **(B)** level of the PM, CB, RB, WB material, and their silage. UPGMA, unweighted pair group method with arithmetic mean; PM, paper mulberry; CB, corn bran; RB, rice bran; WB, wheat bran; PC, PM + CB; PR, PM + RB; PW, PM + WB.

UPGMA hierarchical cluster trees based on the weighted UniFrac distance at the genus (a) and species (b) levels of the PM, CB, RB, WB materials, and their silages are shown in Figures 1A,B. The dominant genera and species in the PM material were *Pantoea* (*Pantoea agglomerans*), *Weissella* (*Weissella paramesenteroides*), and *Enterobacter* (*Enterobacter asburiae*). The dominant genus in CB and WB materials comprised *Oryza sativa Japonica Group Japanese rice*. The dominant genera and species in the RB material were *Methylobacterium* (*Methylobacterium paramesenteroides*) and *Sphingomonas* (*Sphingomonas endophytica*). After ensiling, *Enterobacter* (*E. asburiae*) was present in the PM and PR silages. Furthermore, there was a small proportion of *Clostridium* (*Clostridium tyrobutyricum*) in PR silage. *Lactobacillus* (*Lactobacillus plantarum* and *Lactobacillus* spp.) were the most abundant genera in the PW and PC silages.

**Figure 2:**
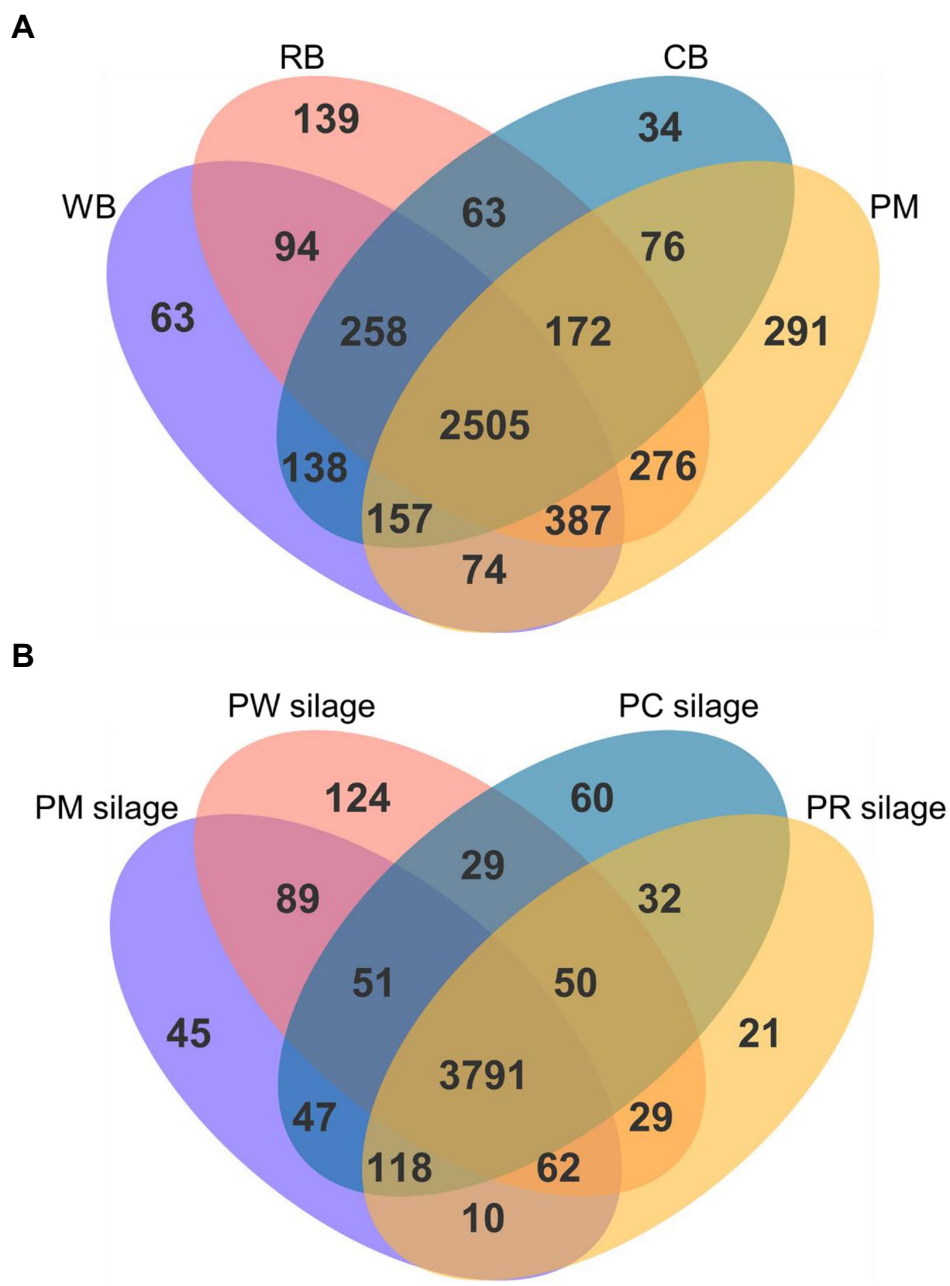
Venn diagram of all the metabolites identified in the PM, CB, RB, WB material **(A)**, and their silage **(B)**. PM, paper mulberry; CB, corn bran; RB, rice bran; WB, wheat bran; PC, PM + CB; PR, PM + RB; PW, PM + WB.

A Venn diagram of the metabolites identified in the PM, CB, RB, and WB materials, as well as their silages, is shown in Figure 2. The number of unique metabolites in PM was 291 (Figure 2A); this value was higher (all *p* < 0.001) than the numbers in CB, RB, and WB (34–139). Compared with PM silage, the number of unique metabolites were higher in PC and PW silages and lower in PR silages (Figure 2B).

**Figure 3:**
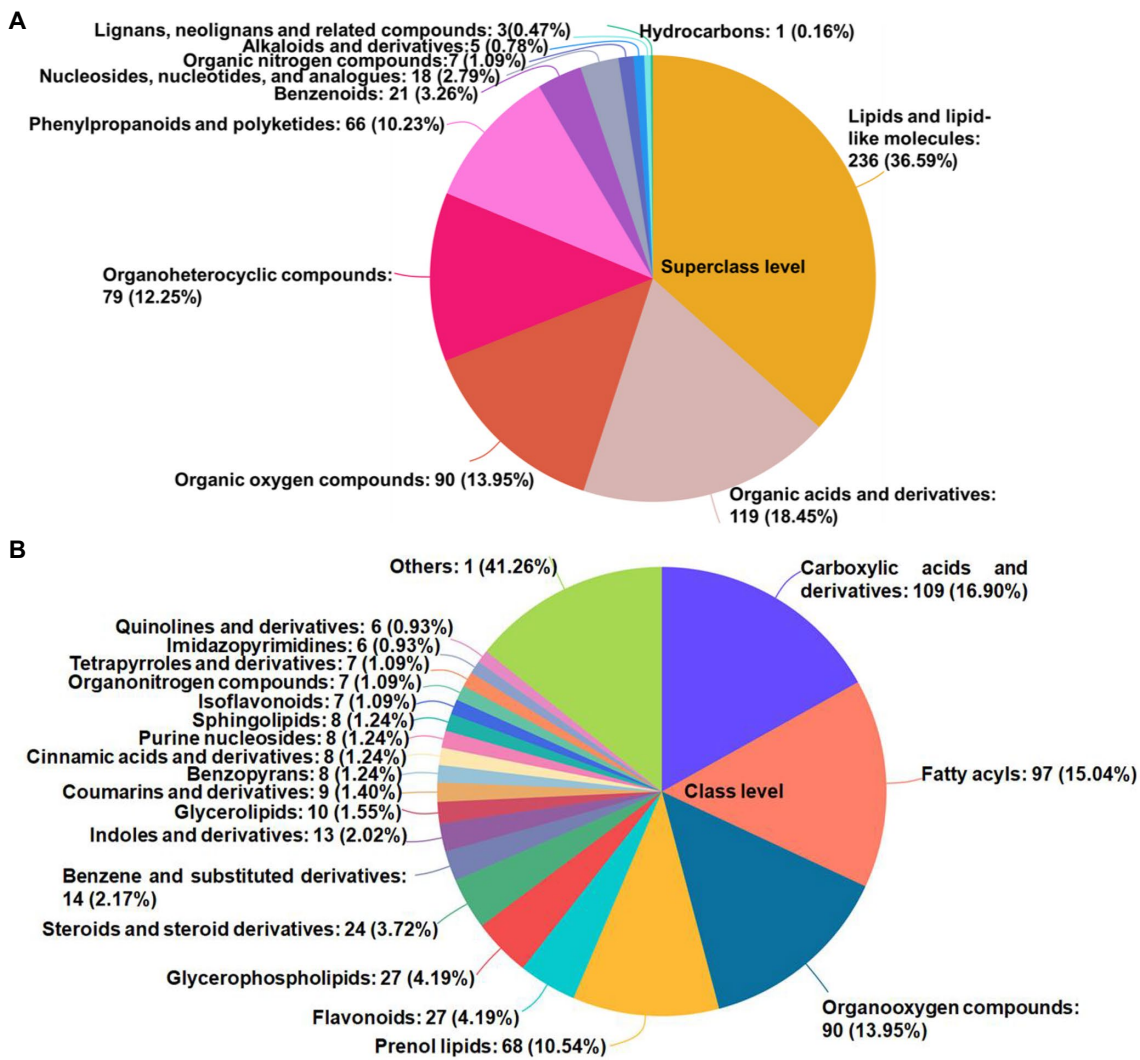
The HMDB compound classification of identified metabolites. The names of the HMDB superclass **(A)** and class **(B)** levels and the percentage of metabolites in order of the number of metabolites were shown. The different colors represented different HMDB classifications, and the area represented the relative proportion of metabolites in this classification. HMDB, human metabolome database.

In total, 645 metabolites—including 11 superclasses (Figure 3A) and 72 classes (Figure 3B); mainly lipids and lipid-like molecules, organic acids and derivatives, organic oxygen compounds, organoheterocyclic compounds, phenylpropanoids and polyketides, and benzenoids—were identified and quantified by querying the HMDB.^1^

The HMDB compound classifications based on the number of metabolites at the superclass level of the PM, CB, RB, and WB materials, as well as their silages, are shown in Table 3. Lipids and lipid-like molecules were the predominant metabolites (27.03–56.90%) in all materials and silages. Compared with materials, silages had significantly greater (*p* < 0.01) contents of lipids and lipid-like molecules. The next most abundant metabolites were organic acids and derivatives, the levels of which were higher (*p* < 0.01) in silages than in materials. The proportions of organic acids and derivatives were similar in PM and PR silages; the highest and lowest proportions were in the PC and PW silages, respectively. The third most-abundant metabolites were organic oxygen compounds, the levels of which were higher (*p* < 0.01) in materials than in silages.

**Table 3:** The HMDB compound classification based on the number of metabolites at the level of superclass of PM, CB, RB, WB material, and their silage.

Metabolites (%)	Material^1^				Silage^2^				*p*
	PM	CB	RB	WB	PM	PC	PR	PW	
Lipids and lipid-like molecules	26.03d	27.23d	34.31c	28.57d	43.27b	56.90a	45.84b	50.10a	<0.001
Organic acids and derivatives	12.93d	14.16c	10.42d	13.87c	18.62b	26.29a	18.63b	28.57a	<0.001
Phenylpropanoids and polyketides	9.72a	8.92c	9.31b	6.19d	7.48d	4.08e	9.90a	8.87c	<0.001
Organoheterocyclic compounds	12.96a	10.13c	11.27b	11.43b	6.80d	4.08f	5.98e	5.56e	<0.001
Organic oxygen compounds	13.77c	18.31a	17.16b	17.14b	5.44e	5.39e	2.72f	6.67d	<0.001
Benzenoids	3.64a	2.82b	1.96d	3.33a	2.72b	2.54c	2.60b	2.62b	<0.001
Lignans, neolignans, related compounds	0.00	0.47d	0.98b	0.48d	0.68c	0.00	1.04	1.16a	<0.001
Organic nitrogen compounds	0.81a	0.47d	0.49d	0.48d	0.68b	0.00	0.52c	0.00	<0.001
Nucleosides, nucleotides, and analogs	2.43c	3.29b	3.92b	1.90d	0.00	0.00	4.69a	0.00	<0.001
Alkaloids and derivatives	1.62c	1.41d	1.97b	1.43d	0.00	0.00	2.08a	0.00	<0.001
Hydrocarbons	0.40b	0.66a	0.00	0.48b	0.00	0.00	0.00	0.00	<0.001
Others	15.69a	12.13b	8.21c	14.70b	14.31b	0.72e	2.05d	0.40e	<0.001

^a–d^Data are means of three samples, means in the same row followed by different letters differ (*p* < 0.05). The number and percentage represent the amount and relative proportion of various metabolites at the superclass classification level in the HMDB for each sample. HMDB, human metabolome database.

1 PM, paper mulberry; CB, corn bran; RB, rice bran; and WB, wheat bran.

2 PC, PM + CB; PR, PM + RB; PW, PM + WB.

**Figure 4:**
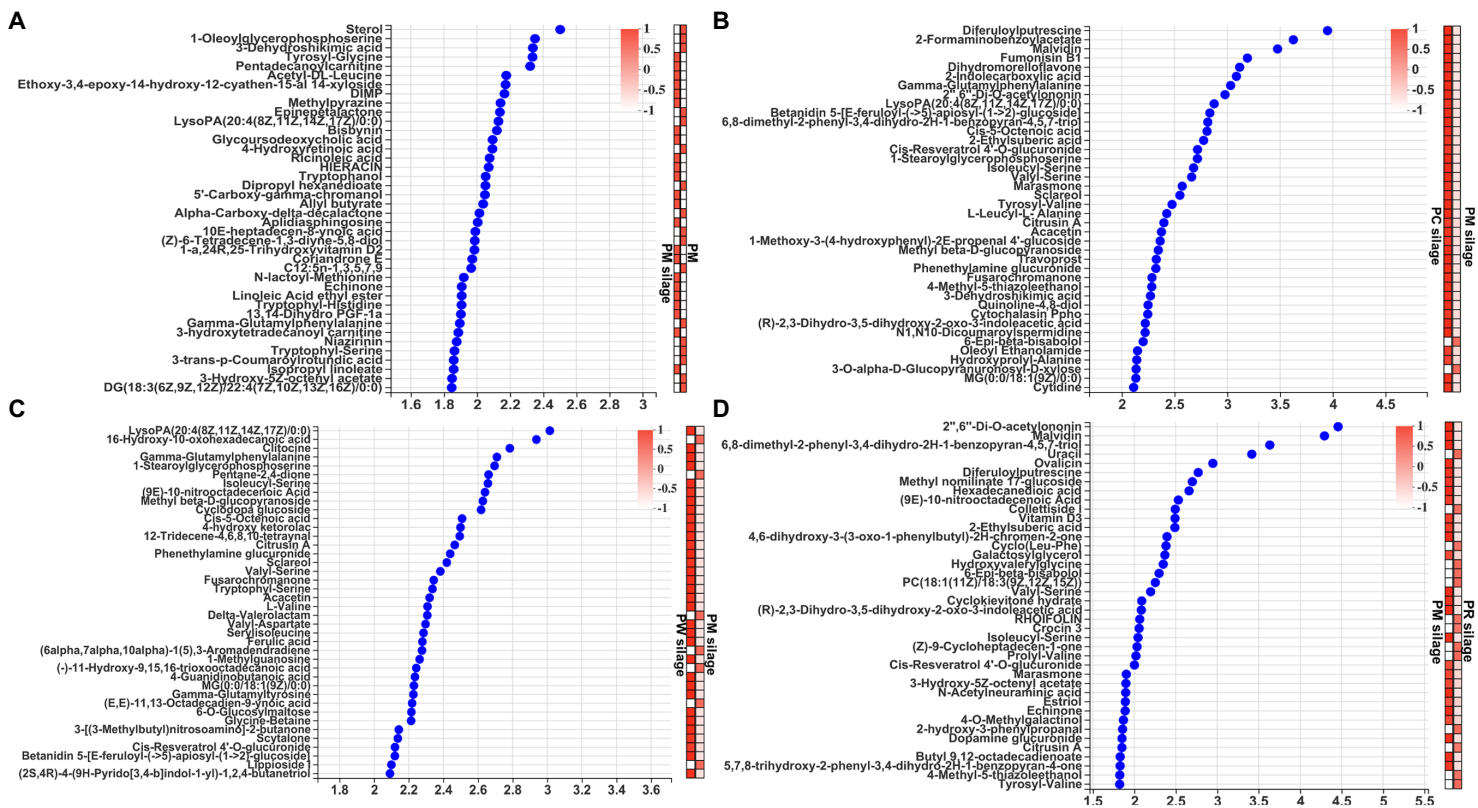
Analysis of the difference of top 40 metabolites based on variable importance in projection (VIP) scores >1 among of the PM, CB, RB, WB material, and their silage: **(A)** the difference of metabolites by PM before and after ensiling; **(B)** the difference of metabolites by PM and PC silages; **(C)** the difference of metabolites by PM and PW silages; and **(D)** the difference of metabolites by PM and PR silages. PM, paper mulberry; CB, corn bran; RB, rice bran; WB, wheat bran; PC, PM + CB.

Analysis of the difference of top 40 metabolites based on VIP scores > 1 of the OPLS-DA are shown in Figure 4. The levels of essential amino acids (Leu, Trp, Met, and Phe) and semi-essential amino acids (His and Tyr) were higher (Figure 4A) in PM silage than in PM (all *p* < 0.001). Furthermore, the contents of low-grade aromatic esters (allyl butyrate and α-carboxy-delta-decalactone) and fatty acids (linoleic acid ethyl ester and isopropyl linoleate) were significantly increased (all *p* < 0.001) in PM silage, compared with PM. PC silage had higher (all *p* < 0.001) aromatic compound (sclareol), amino acid (Ser and Phe), fatty acid (indole carboxylic acid), and antimicrobial contents (benzopyran) than did PM silage (Figure 4B). PW silage had significantly increased (all *p* < 0.001) contents of amino acids (Ser, Val, Asp, and Ile), a fatty acid (10-nitrooctadecenoic acid), aromatic compound (sclareol), and antimicrobial (ferulic acid) than did PM silage (Figure 4C). PR silage had significantly decreased (all *p* < 0.001) contents of amino acids (Ser and Val), a fatty acid (10-nitrooctadecenoic acid), aromatic ester (ethylsuberic acid), and antimicrobial (acetylononin) than did PM silage (Figure 4D).

**Figure 5:**
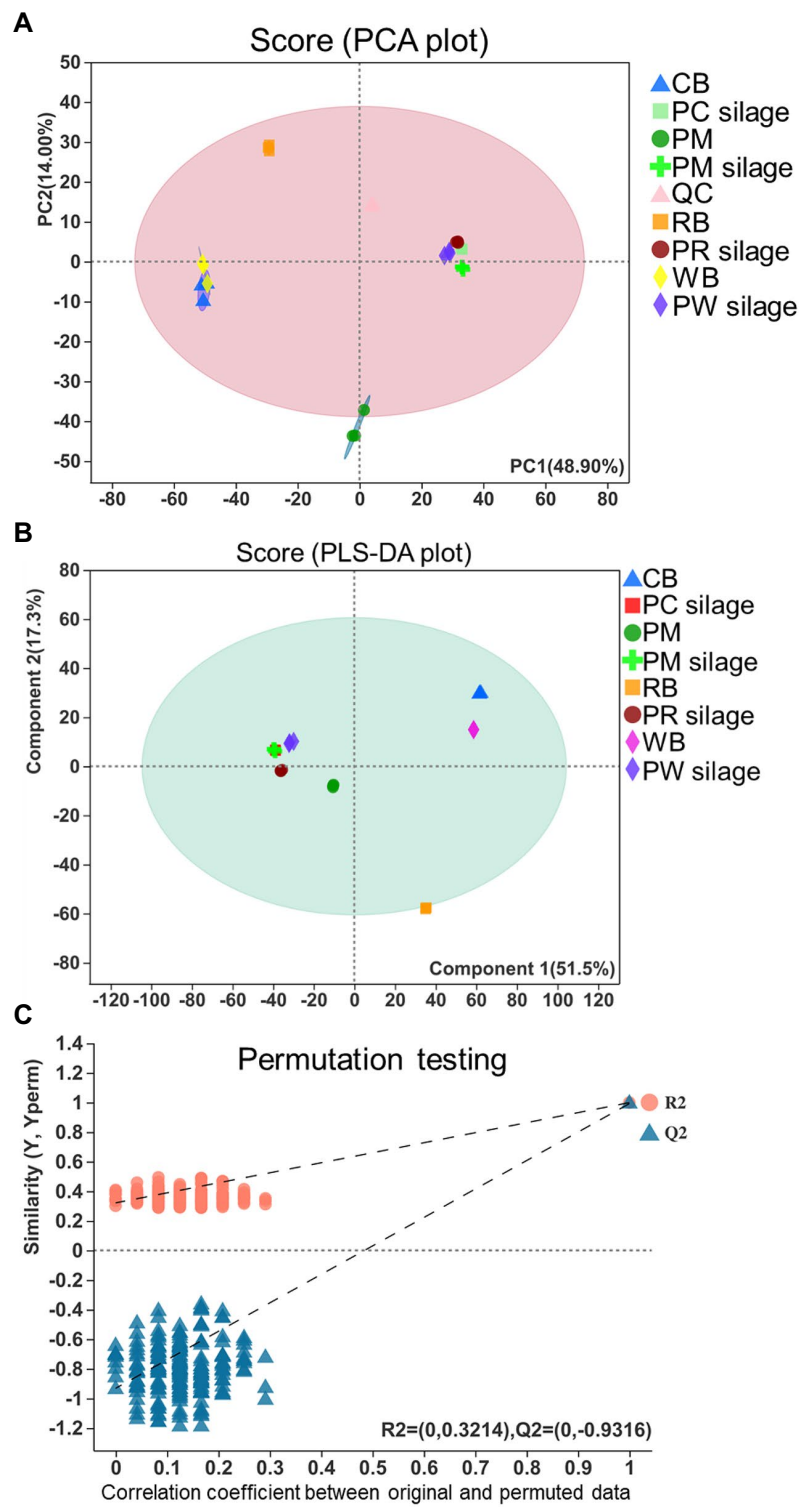
PCA and PLS-DA score plots resulted from the metabolites PM, CB, RB, WB material, and their silage **(A,B)** and permutation tests of the PLS-DA model indicate the absence of over-fitting in the model **(C)**. PCA, principal component analysis; PLS-DA, partial least squares-discriminate analysis; PM, paper mulberry; CB, corn bran; RB, rice bran; WB, wheat bran; PC, PM + CB; PR, PM + RB; PW, PM + WB; QC, quality control.

The PCA and PLS-DA of metabolic profiles in PM, CB, RB, and WB materials, as well as their silages, are shown in Figure 5. According to PCA (Figure 5A), the metabolites in PM, PC, PR, and PB silages were separated by PC1, which explained 48.90% of the variation. The altered metabolites in PM, PC, PR and PB silages were separated by the first component of the PCA model; the metabolites in PR silage were separated by the second component. The multivariate analysis PLS-DA was better able to distinguish PM silage from PC, PR, and PW silages (Figure 5B). A chance permutation test (R^2^Y-intercept = 0.3214, and Q^2^-intercept = −0.9316) showed no overfitting (Figure 5C).

**Figure 6:**
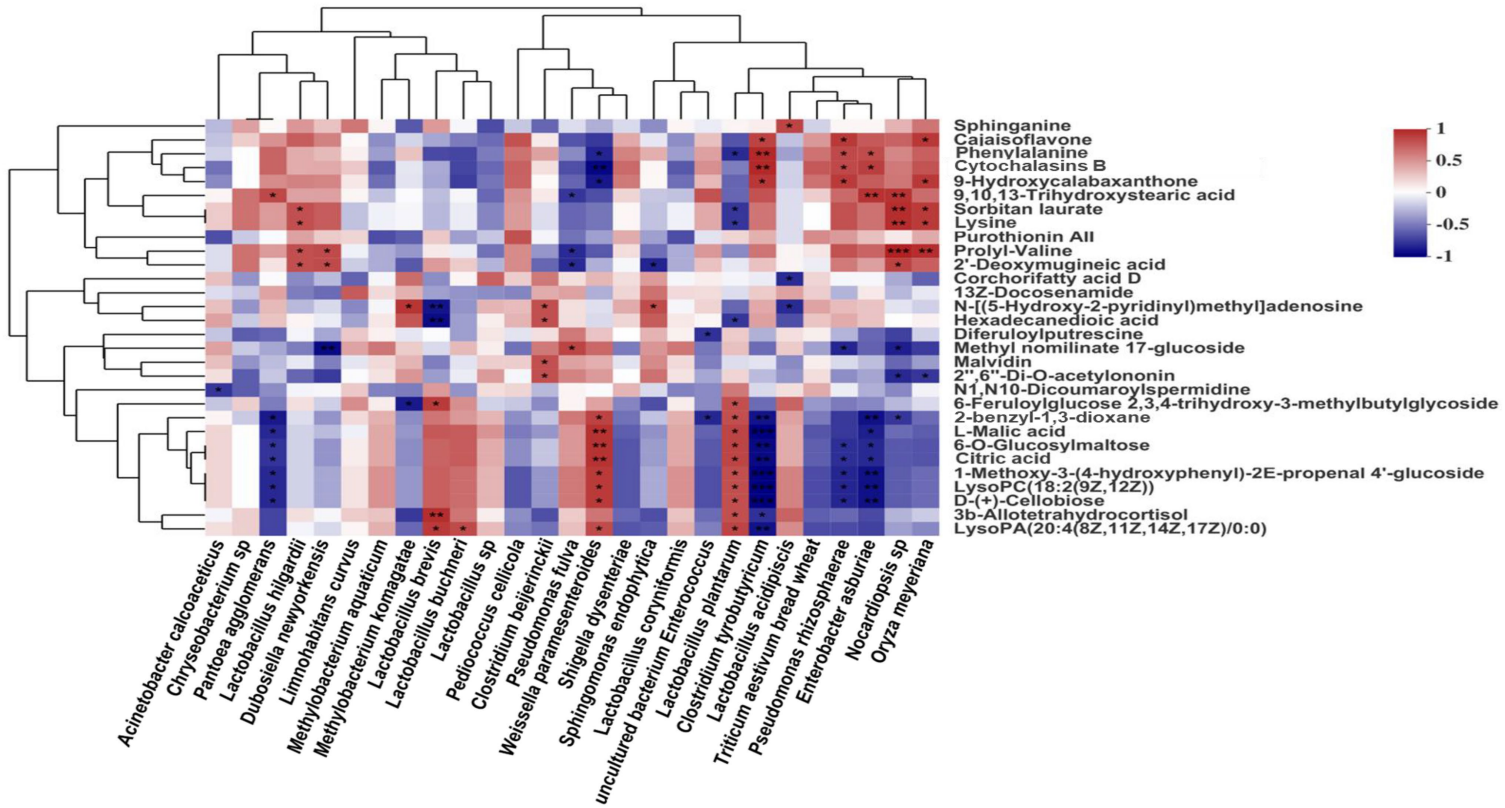
Spearman’s correlations between main bacteria species and differentially presented metabolites in silage. Values of *p* are shown as *0.01 < *p* < 0.05 and **p < 0.01. The corresponding value of the heat map is the Spearman’s correlation coefficient *r*, which ranges between −1 and 1, *r* < 0 indicates a negative correlation (blue), *r* > 0 indicates a positive correlation (red).

Spearman correlations between the main bacterial species and metabolites in silages are shown in Figure 6. *L. plantarum* and *W. paramesenteroides* were positively (all *p* < 0.05) correlated with the presence of L-malic acid, citric acid, and D-(+)-cellobiose, whereas *C. tyrobutyricum* and *E. asburiae* showed the opposite pattern.

## Discussion

### Fermentation Characteristics of PM Silage

In the tropics, with the increase in population and decrease in arable land, the competition between humans and animals for food and feed has become increasingly prominent. Conventional feeds such as forage crop, grass, and grain cannot meet the needs of ruminants. In response to the shortage of feed in the dry season, natural feed resources such as WP are under active development for use in animal production. PM is a popular WP in tropical regions and has good livestock palatability. Its nutritional value is similar to the nutritional value of legume grasses, and it has the reputation of woody alfalfa (Dong et al., 2020). In this study, the CP content of fresh stems and leaves of PM was 24% of DM, indicating that it can be used as a source of high-protein livestock feed. Because of the high moisture of WP, like other herbaceous forages, it is generally difficult to make high-quality silage (Cai et al., 1999; Du et al., 2021b). As shown in Table 1, the moisture content of PM was >80%, indicating the need for adjustment during the silage process. In addition, WSC, LAB, and LBC affect silage fermentation (Cai et al., 1999). The addition of 30% CB, RB, and WB to PM reduced the moisture content to <65%, within the ideal range (60%–70%) for preparing high-quality silage (McDonald et al., 1991). A suitable moisture content inhibits fermentation by harmful clostridia (Wan et al., 2021), while the addition of exogenous additives increases the fermentation substrate for LAB and alleviates LBC, thereby improving silage fermentation quality (Cai et al., 2020).

In this study, the addition of CB and WB improved silage fermentation quality (Table 2). However, contrary to our expectations, the addition of RB resulted in butyric acid fermentation and proteolysis in PM silage, and the underlying mechanism of this process is unclear. RB is rich in EE and can promote the proliferation of clostridia during ensiling (Kiyoshi and Choseki, 1975), and lead to protein degradation. Clostridia are commonly found in anaerobic environments such as paddy soils, and their spores are incorporated into bran during rice harvesting and rice production. Generally, the anaerobic environment formed by irrigation in paddy fields during rice cultivation is much higher than that in upland field, such as corn and wheat cultivation (Du et al., 2021b). Therefore, we speculate that absolute anaerobic microorganisms such as *Clostridium* species in paddy fields are higher than those in ordinary upland field. In this study, the results of SMRT sequencing confirmed the presence of *Clostridium* spp. in RB, whose silage contained a large amount of NH_3_-N. Therefore, RB will affect the fermentation quality of PM silage, resulting in nutrient loss.

### Microbiota of Material and Silage

Various microorganisms are present in the stems and leaves of forage crops, grasses, and other plants. Their community structures, species diversities, and metabolic functions are important determinants of silage fermentation (McEniry et al., 2010; Guan et al., 2018; Keeling et al., 2019). In this study (Figure 1A), the genera *Pantoea*, *Enterobacter*, and *Weissella* were the major epiphytic bacteria in PM material. *Pantoea* is a Gram-negative pathogen, present in various environments; however, it is uncommon in silage. *Pantoea* decompose and metabolize organic compounds, leading to the deterioration of fermented food and feed (Avila and Carvalho, 2020). *Enterobacter* and *Weissella* are common microorganisms in silage. *Enterobacter* is a Gram-negative genus that dominates the bacterial community of fresh forage. In the early stage of silage fermentation, *Enterobacter* and *Weissella* compete with LAB, causing nutrient loss. However, as fermentation progresses, their proliferation is inhibited; they are subsequently replaced by LAB (Heron et al., 1993; Pahlow et al., 2003). In the silage process, D-lactic acid and carbon dioxide are produced, influencing the proportion of lactic acid isomers and increasing the loss of DM in silage (Cai et al., 1998). *Oryza sativa* Japonica Group Japanese rice was significantly enriched in the CB and WB materials; *Methylobacterium*, *Sphingomonas*, and *Clostridium* were predominant in the RB materials. Except for *Clostridium*, these bacteria are rare in silage materials, and their influence on silage fermentation is unclear. They are generally considered to be unfavorable bacteria for silage production (Du et al., 2021b). *Clostridium* affects the fermentation of silage by degrading proteins and causing nutrient loss.

After ensiling, *Enterobacter* was predominant in the PM and PR silages; it competed with LAB for nutrients in the anaerobic environment (Santos et al., 2016). The genus *Clostridium* was also a major taxon in PR silage. The addition of RB may increase the EE and clostridial spore contents, thus promoting the proliferation of *Clostridium* spp. (Queiroz et al., 2018). The effects of adding CB and RB to PM silage on the moisture and fermentation substrate contents may promote the growth of LAB and inhibit the growth of harmful bacteria. Indeed, the dominant genus in both PC and PW silages was *Lactobacillus*. Notably, the epiphytic microbial community in the materials comprised <1% of genera, indicating that these bacteria were rapidly suppressed after ensiling.

The dominant species in the materials were *P. agglomerans*, *E. asburiae*, and *W. paramesenteroides* in PM; they were *Methylobacterium aquaticum* and *Sphingomonas endophytica* in RB (Figure 1B). Except for *W. paramesenteroides*, these bacteria are considered harmful and presumed to reduce silage fermentation quality. *W. paramesenteroides* is a heterofermentative LAB typically found in silage. In the initial stage of fermentation, it grows vigorously and produces carbon dioxide, creating an anaerobic environment that promotes fermentation by other acid-resistant LAB. *L. plantarum* was predominant in high-quality PC and PW silage, whereas *E. asburiae* and *C. tyrobutyricum* were predominant in low-quality PM and PR silage. Such changes in microbial population structure indicate that these microbes are determinants of silage fermentation. The preparation of silage affects fermentation quality, and the microbial community in silage is important for the physiology and health of livestock.

### Metabolomic Profiles in the Material and Silage

The combination of microbiome and metabolomics profiling can provide microbial community and metabolite information; it can also expand the functions of silage (Xu et al., 2019). The materials before ensiling were aerobic but not acidic, and the high moisture content of fresh PM promoted the growth of microorganisms with corresponding metabolite production. However, the low moisture content of CB, RB, and WB suppressed this growth and metabolite production (Figure 2A).

Microorganisms are typically sensitive to anaerobic and acidic environments (Du et al., 2021b). Some Gram-negative aerobic bacteria have thin cell walls and weak acid resistance; they are rapidly inactivated by the anaerobic and acidic silage fermentation environment. However, LAB grow rapidly and metabolize both amino acids and carbohydrates in anaerobic and acidic environments. The increased LAB population in PC and PW silages increased their metabolite contents (Figure 2B). In the PM and PR silages, *Enterobacter* and *Clostridium* spp. were more abundant than LAB, reducing the metabolic capacity of LAB and their corresponding metabolite production.

### HMDB Compound Classification of Identified Metabolites

Several functional organic acids (Figure 3A) and derivatives (carboxylic acids and derivatives) and flavoring agents (benzoic acids and derivatives; Figure 3B) were detected in the PM and mixture silages. These metabolites were mainly lipids and lipid-like molecules, organic acids and derivatives, and organic oxygen compounds (Table 3). Lipids and lipid-like molecules comprise a superclass of organic compounds, including fatty acids and their derivatives. LAB produce multiple metabolites of fatty acids during fermentation (Sun et al., 2012). Fatty acids improve the growth and milk fat components of dairy cattle, thereby improving their health. The greater proportion of LAB in PC and PW silages led to higher fatty acid content than in other silages. In the PM and PR silages, the harmful bacteria inhibited the growth of LAB, suppressing fatty acid synthesis. Organic acids (e.g., fumaric acid, malic acid, citric acid, and succinic acid) in silage can increase livestock performance and feed efficiency; they are used as substitutes for feed antibiotics (Ke et al., 2017). In this study, the greater proportion of LAB in PM and PR silages increased production of organic acids and derivatives, thereby improving silage fermentation quality. Organic oxygen compounds comprise a superclass of organooxygen compounds, including carbohydrates and carbohydrate conjugates. Compared with the materials, the organic oxygen compound content was significantly reduced in the silages. This is because during the ensiling process, *Lactobacillus* and *Clostridium* use glycosyl compounds to produce lactic acid and spores, thereby decreasing the carbohydrate content.

### VIP Scores of the Top 40 Metabolites in Material and Silage

Microbial fermentation products including organic acids and 1,2-propanediol have been used as indicators of silage fermentation quality and aerobic stability (Xu et al., 2019; Cai et al., 2020). However, these indicators cannot fully reflect the complex metabolic processes of silage. Metabolomics analysis enables evaluation of the fermentation, nutritional, and functional characteristics of silage (Guo et al., 2018). The quality of feed protein depends on the balance among multiple amino acids (Tedeschi et al., 2015). In this study (Figures 4A–C), greater abundances of Leu, Try, Met, Phe, Ser, His, Val, Asp, and Ile were associated with better ensiling quality in PC and PW silages, compared with PM silage. The increased amino acid contents in PC and PW silages may be related to the unique sugar fermentation and amino acid biosynthesis pathways of LAB, such as *L. plantarum*. These data indicate that the high-quality PM silage generated by addition of CB and WB suppresses protein degradation. The opposite pattern was observed in PR silage (Figure 4D). *Clostridium* spp. in PR silage could promote the degradation of protein to NH_3_-N. Feeding silage can provide fatty acids to ruminants, which can increase the content of unsaturated fatty acids in milk and reduce the ratio of saturated fatty acids; it also increases the levels of essential fatty acids. Therefore, the fatty acids produced by LAB in silage can be used to promote animal health and welfare (Salsinha et al., 2018). The CB and WB had significantly increased levels of unsaturated fatty acids (linoleic acid, isopropyl linoleate, indole carboxylic acid, and nitro-octadecenoic acid), which exert an antioxidant effect, eliminate free radicals, and reduce cancer incidence. However, RB inhibits the synthesis of unsaturated fatty acids. Some of the aromatic compounds detected are sweeteners or flavoring agents. *Lactobacillus plantarum* in PC and PW silages uses sugars to produce aromatic compounds (allyl butyrate, α-carboxy-delta-decalactone, sclareol, and ethylsuberic acid), improving feed flavor and quality. However, *Clostridium* spp. in PR silage inhibited aromatic compound biosynthesis, explaining its poor aroma. In addition, antimicrobial metabolites were detected. Benzopyran and ferulic acid exhibit antioxidant properties; they promoted the proliferation of LAB and inhibited harmful bacteria in PC and PW silages. However, acetylononin is an inhibitor of isoflavonoid o-glycosides, and it may inhibit organic compound synthesis.

### Metabolite Profiles of the Material and Silage

In PC and PW silages, the addition of CB and WB caused *Lactobacillus plantarum* to predominate, thereby altering metabolite levels. Compared with PM silage, the addition of RB did not affect the bacterial community, likely because *Enterobacter* and *Clostridium* were predominant and led to similar metabolite profiles (Figures 5A,B). The results of permutation testing supported this finding (Figure 5C).

### Correlations Between Main Bacterial Species and Metabolites

Spearman correlation analysis regarding the main metabolites of bacteria in silage showed that during fermentation, metabolites were positively correlated with LAB and negatively correlated with other bacteria (Xu et al., 2019). Citric acid and L-malic acid are key intermediates in the citric acid cycle; they were produced by most LAB species LAB can use D-(+)-cellobiose as a substrate for energy production, accelerating their growth and inhibiting the growth of yeasts, molds, and *Clostridium* and *Enterobacter* spp. (Ke et al., 2017). L-Malic acid and citric acid may suppress protein hydrolysis by reducing pH, which enhances mixed ruminal microorganism fermentation *in vitro* and improves animal performance (Wang et al., 2009). In addition, L-malic acid and citric acid are antioxidants and inhibit lipoxygenases. In this study (Figure 6), *L. plantarum* and *W. paramesenteroides* were positively correlated with the L-malic acid, citric acid, and D-(+)-cellobiose contents. In contrast, *C. tyrobutyricum* and *E. asburiae* were negatively correlated with these metabolites. Overall, our findings indicate that the silage microbiota and metabolome are closely related and exhibit important interactions. Succession of the microbial population affects metabolite levels; changes in metabolite levels also influence the microbial population. The metabolic activities of microorganisms affect silage fermentation quality and have important roles in the production of healthy livestock.

## Conclusion

To explore the fermentation of WP silage, PacBio SMRT sequencing and untargeted metabolomics methods were used to study the microbiota–metabolome interaction in WP silage treated with bran. CB and WB accelerated the succession of dominant bacteria to LAB in silage; they increased the contents of amino acids, unsaturated fatty acids, citric acid, L-malic acid, and other flavoring agents, thereby improving silage flavor and quality. RB promoted butyric acid fermentation by clostridia, thus increasing the levels of volatile fatty acids and NH_3_-N, which resulted in poor fermentation. The combination of microbiome profiling and metabolomics enables analysis of the interactions of microorganisms, metabolites, and fermentation mechanisms during silage fermentation, providing important scientific insights for the development and use of WP resources. Therefore, PM is a potential high-protein feed resource for ruminants, which can not only alleviate the shortage of feed, but also play an important role in producing low-cost feed and increasing farmers’ income.

## Data Availability Statement

The datasets presented in this study can be found in online repositories. The names of the repository/repositories and accession number(s) can be found at: https://www.ebi.ac.uk/metabolights/search MTBLS4443.

## Author Contributions

ZD, YC, and FY: conceptualization, methodology, and writing—original draft. ZD and YC: formal analysis. ZD, LS, and YL: sample processing. ZD and LS: sequencing. ZD and YL: collection of metadata. ZD, LS, YL, FY, and YC: writing—reviewing and editing. ZD, LS, and YC: visualization. YC and FY: supervision. FY: funding acquisition. All authors contributed to the article and approved the submitted version.

## Funding

This study was supported by the National Key R&D project “Processing technology research and demonstration of high-quality forage silage and molded product” (2017YFD0502102) funded by the Ministry of Science and Technology, China, and the JIRCAS Visiting Research Fellowship Program 2019–2020 of the Japan International Research Centre for Agricultural Sciences, Japan.

## Conflict of Interest

The authors declare that the research was conducted in the absence of any commercial or financial relationships that could be construed as a potential conflict of interest.

## Publisher’s Note

All claims expressed in this article are solely those of the authors and do not necessarily represent those of their affiliated organizations, or those of the publisher, the editors and the reviewers. Any product that may be evaluated in this article, or claim that may be made by its manufacturer, is not guaranteed or endorsed by the publisher.

## Figures and Tables

**Table:** 

PM	Paper mulberry
SDGs	Sustainable development goals
WSC	Water-soluble carbohydrate
CB	Corn bran
RB	Rice bran
WB	Wheat bran
PacBio	Pacific Biosciences
SMRT	Single-molecule real-time
FM	Fresh matter
DM	Dry matter
OM	Organic matter
CP	Crude protein
EE	Ether extract
NDF	Neutral detergent fiber
ADF	Acid detergent fiber
ADL	Acid detergent lignin
NH_3_-N	Ammonia nitrogen
LBC	Lactic acid buffer capacity
LAB	Lactic acid bacteria
cfu/g	Colony-forming unit per gram
DNA	Deoxyribonucleic acid
PCR	Program control register
OTU	Operational taxonomic unit
VIP	Importance in projection
PCA	Principal component analysis
PLS-DA	Partial least-square discriminant analysis
OPLS-DA	Orthogonal partial least squares discriminant analysis

## Footnotes

^1^ http://www.hmdb.ca/
